# Molecular Imaging of Fluorinated Probes for Tau Protein and Amyloid-β Detection

**DOI:** 10.3390/molecules25153413

**Published:** 2020-07-28

**Authors:** Sarah K. Yeo, Yurii Shepelytskyi, Vira Grynko, Mitchell S. Albert

**Affiliations:** 1 Biology Department, Lakehead University, Thunder Bay, ON P7B 5E1, Canada; skyeo@lakeheadu.ca; 2 Chemistry and Materials Science Program, Lakehead University, Thunder Bay, ON P7B 5E1, Canada; yshepely@lakeheadu.ca (Y.S.); vgrynko@lakeheadu.ca (V.G.); 3 Thunder Bay Regional Health Research Institute, Thunder Bay, ON P7B 6V4, Canada; 4 Chemistry Department, Lakehead University, Thunder Bay, ON P7B 5E1, Canada; 5 Northern Ontario School of Medicine, Thunder Bay, ON P7B 5E1, Canada

**Keywords:** molecular imaging, Alzheimer’s disease, MRI, PET, fluorescence, fluorinated molecular probes, amyloid beta imaging, neurofibrillary tangle imaging

## Abstract

Alzheimer’s disease (AD) is the most common form of dementia and results in progressive neurodegeneration. The incidence rate of AD is increasing, creating a major public health issue. AD is characterized by neurofibrillary tangles (NFTs) composed of hyperphosphorylated tau protein and senile plaques composed of amyloid-β (Aβ). Currently, a definitive diagnosis of AD is accomplished post-mortem. Thus, the use of molecular probes that are able to selectively bind to NFTs or Aβ can be valuable tools for the accurate and early diagnosis of AD. The aim of this review is to summarize and highlight fluorinated molecular probes that can be used for molecular imaging to detect either NFTs or Aβ. Specifically, fluorinated molecular probes used in conjunction with ^19^F MRI, PET, and fluorescence imaging will be explored.

## 1. Introduction

Alzheimer’s disease (AD) is the most common form of dementia and is characterized by a progressive loss of cognitive function. It accounts for an estimated 60% to 80% of all cases of dementia [1]. As of now, it is estimated that 5.8 million Americans aged 65 years and older have AD. This number is estimated to rise to 7.1 million by 2025 and 13.8 million by 2050, which may hinder developments to prevent or cure AD [1]. Neurofibrillary tangles (NFTs) containing hyperphosphorylated and aggerated tau proteins and senile plaques containing amyloid-β (Aβ) within the brain represent the pathological characteristics of Alzheimer’s disease (AD). The deposit of NFTs in the hippocampus and entorhinal cortex is correlated with the severity of cognitive decline. Similarly, amyloid plaques have been found to accumulate within the isocortex, although the progression of the deposition is less predictable than NFTs [2]. The amyloid cascade hypothesis suggests that the deposition of Aβ is the first change to the brain during AD, which leads to senile plaque formation, followed by NFTs and neuronal cell death [3]. However, only tau NFTs correlate with the degree of cognitive dysfunction [4]. Currently, AD is diagnosed through a series of cognitive tests done by a physician, as well as obtaining a medical and family history from the individual [1]. However, a definitive diagnosis is accomplished histopathologically through the observation of pathological characteristics such as Aβ plaques and NFTs in post-mortem brain tissues [5]. Thus, the need for a noninvasive tool to identify the presence of Aβ plaques or NFTs can prove to be useful in the accurate diagnosis of AD. 

The use of molecular probes to image NFTs and Aβ plaques has mainly focused on the use of positron emission tomography (PET) [6]. This medical imaging technique utilizes radiolabeled probes that emit positrons. Once emitted, positrons travel over a short range in the body and annihilate with an electron. As a result of annihilation, two gamma quants are emitted and used to produce an image [7,8,9]. The main advantage of this approach is the extremally high sensitivity and specificity of PET tracers [6]. However, PET has a low spatial resolution [10], which may make it difficult in analyzing pathological characteristics in small structures such as the hippocampus. Additionally, PET tracers require fast imaging right after synthesis and purification due to the respective radioactive decay. 

Magnetic resonance imaging (MRI) is another medical imaging modality used for studying AD. MRI has been used to measure water diffusion changes in the brain, AD-associated changes in brain perfusions, structural changes, and brain atrophy and study the effects of AD on the functional connectivity network of the brain [6,11,12]. Despite the high spatial resolution of MRI, molecular imaging using this medical imaging modality is still under development due to the low sensitivity of MRI [6]. The recent progress in the field of MRI AD molecular imaging has been connected with the development of fluorinated molecular probes. Fluorine-19 (19F) has a high natural abundance (~100%) and a high gyromagnetic ratio. Additionally, there is a lack of endogenous 19F in living organisms. These three factors cause the reasonable signal-to-noise ration of 19F MRI images [13]. MRI can potentially be used for molecular imaging scans that have a higher spatial resolution than PET, are less cost-effective, and do not use ionizing radiation. MRI used for molecular imaging is advantageous, since the probes can be stored long-term before use [14]. 

Another upcoming technique used for molecular imaging is fluorescence imaging with near-infrared light. The fluorescence emission of each probe used should be within 650 and 900 nm to achieve an adequate penetration depth and high sensitivity. The advantage of optical imaging includes the low cost, real-time imaging with the option of multitargets, wide availability, and no radioactivity [15]. Notwithstanding the application potential of fluorinated molecular imaging probes for AD detection, comprehensive reviews on fluorinated molecular probes are lacking, leaving a strong demand for an article to fill this gap. The purpose of this review is to provide an update on the recent developments and advances in fluorinated molecular probes for imaging AD. A complete review of fluorinated molecular probes that have been developed will facilitate the translation from the laboratory to future clinical applications.

## 2. 19F MRI Molecular Probes for AD Detection

### 2.1. Amyloid Beta Imaging

**Figure 1:**
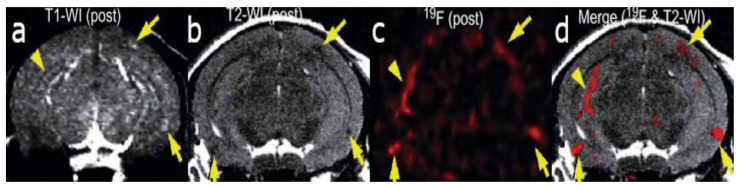
In vivo detection of amyloid plaques (indicated by arrowheads and arrows) in the brain of a Tg2576 mouse after injection with (E,E)-1-fluoro-2,5-bis(3-hydroxycarbonyl-4-hydroxy)styryl-benzene (FSB) [14]. (**a**) T1-weighted 1H imaging of the mouse brain. (**b**) T2-weighted image of the same slice indicating the position of the amyloid-beta plaques. (**c**) 19F MRI image of the mouse brain after FSB injection. (**d**) 19F-FSB image overlaid on top of the high-resolution T2-weighted 1H image. Images are reprinted with permission from the publisher [14].

The deposition of amyloid-β (Aβ) plaques within the brain has been considered the initiating event in the development of Alzheimer’s disease (AD) and occurs before clinical symptoms emerge [16,17]. Thus, the use of a probe that can selectively bind to Aβ within the brain can be a valuable tool in monitoring AD progression, as well as the evaluation of responses to anti-amyloid therapies. The first study to detect Aβ in amyloid precursor protein (APP) transgenic mice using 19F-MRI was reported using an amyloidophilic monofluorinated bis-styryl-benzene ligand, (E,E)-1-fluoro-2,5-bis(3-hydroxycarbonyl-4-hydroxy)styryl-benzene (FSB), obtained with 9.4-T MR imaging (Figure 1). Although the sensitivity of FSB was low and required images averaging over two hours, the results indicated that additional compounds can be fluorinated to obtain similar amyloid-targeted probes with even better sensitivities to detect AD [14]. Since then, several fluorinated compounds have been used to detect Aβ in vivo. Several polyfluorinated bisstyryl-benzenes have been investigated for Aβ detection by Flaherty et al. [18]. Compounds with a tetrafluorophenyl core increased their binding affinity to Aβ compared to their monofluorophenyl counterparts due to the more electropositive tetrafluorophenyl core. Additionally, bisstyryl-benzenes with para-substitutions have a higher affinity to Aβ than those with meta-substitutions. Specifically, (E,E)-1,2,4,5-tetrafluoro-3,6-bis(4-hydroxy)styryl-benzene and (E,E)-1,4-bis(4-trifluromethoxy)styryl-benzene were shown to have increased Aβ binding affinities (Kd) of 0.030 ± 0.001 and 10 ± 1 nM and Aβ plaque binding specificities (signal/noise ratio) of 10 ± 1.3 and 8.6 ± 0.2, respectively. These specificities show the relative affinity of a compound to Aβ plaques compared to normal brain tissue. Both compounds also have the ability to pass the blood-brain barrier, showing the necessary characteristics for Aβ detection. However, there are no available results showing the detection in vivo [18].

Trifluoromethoxy-benzylated compounds such as 2-(4’-dimethylaminostyryl)-6-(2-([3’-trifluoromethoxy](benzylamino)ethoxyethoxy)benzoxazole (TFMB-2Et) and 2-(4’-dimethylaminostyryl)-6-(2-(3-trifluoro-methoxy)benzylaminoethoxyethoxyethoxy)benzoxazole (TFMB-3Et) have been investigated as candidate probes for amyloid imaging in vivo and have stronger 19F signals than FSB [19]. However, 19F NMR signals are sensitive to the environmental conditions of tissues. Brain tissue was found to reduce the signal intensity and inhibit amyloid detection by affecting the 19F NMR signal. This may be due to the high proportion of lipid components within the brain that trap the ligands containing hydrophobic 19F. To overcome this, the hydrophilicity of the probes can be increased for the efficient detection of amyloids. However, the level of hydrophilicity can affect the rate of passage of the compound across the blood-brain barrier. Ensuring the optimization of the probe’s hydrophilicity while also maintaining sufficient brain permeability is essential for amyloid MR imaging of the brain [19].

Curcumin derivatives (19F-containing) have been investigated for the detection of Aβ deposition using 19F MRI [20,21]. Curcumin, derived from the turmeric plant, has anti-inflammatory and antioxidant properties and has been found to suppress amyloid effects [22,23]. Several studies have confirmed its ability to pass the blood-brain barrier, bind Aβ plaques, and reduce the levels of existing plaques within the brain [20,22,24], suggesting its use as a possible probe to detect Aβ deposition. 19F-containing curcumin derivatives such as 1,7-bis(4-hydroxy-3-trifluoromethoxyphenyl)-4-methoxycarbonylethyl-1,6-heptadiene-3,5-dione (FMeC1) have been developed and shown to pass the blood-brain barrier and have an affinity to Aβ plaques in a live mouse model of AD [20]. It is important to note that curcumin and its derivatives exhibit keto-enol tautomerism, where only the enol form can bind to Aβ aggregates and fibrils. FMeC1 exists in the keto form in physiological buffers and shifts to the enol form when bound to Aβ aggregates [25]. Using a 7.0-T MR scanner, the in vivo detection of Aβ depositions was accomplished using FMeC1 in a mouse model of AD [20]. Since curcumin is amphipathic and requires high concentrations of detergents to pass the blood-brain barrier, it makes administration by intravenous injection lethal and human trials unattainable [26]. Thus, McClure et al. used inhalation to deliver an aerosolized solution of perfluoro curcumin analog (FMeCl) to the brain [21]. The compound was prepared in the same solution used for intravenous injection; however, the suspension was aerosolized with a center-flow atomizer, diluted with air, and delivered to live mice by nose-only inhalation. Using this method, FMeCl reached concentrations in the brain and was detectable by 19F NMR at 300 MHz [21]. 

**Figure 2:**
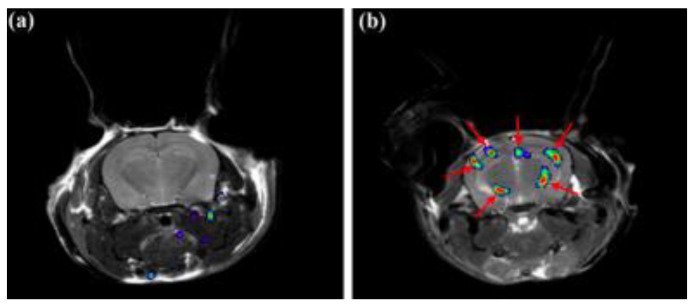
19F MRI signals of bovine serum albumin@F-functionalized graphene quantum dots (BSA@FGQDs) in normal (**a**) and Alzheimer’s disease (AD) (**b**) mice overlaid on top of a high-resolution T1-weighted proton MRI image [27]. Arrows indicate the detection of in vivo amyloid plaques. Images are reprinted with permission from the publisher [27].

In order to monitor the amyloid aggregation, a previous study used F-functionalized graphene quantum dots (FGQDs) in conjunction with 19F MRI and fluorescence spectroscopy to monitor the intermediate species involved. Bovine serum albumin (BSA) was capped onto the FGQDs and was able to bind to amyloid intermediates by π-π stacking interactions and carboxyl/amino groups. The probe was effective in monitoring in vitro amyloid aggregation with a higher specificity and selectivity than thioflavin dye and was suitable for in vivo MRI detection, since it was able to pass the blood-brain barrier and contains 19F (Figure 2) [27].

Recently, the possibility of using 2-(*p*-(trifluoromethyl)phenyl)-1,3-benzothiazol for the detection of AD using ex vivo brain tissue at 3T MRI has been demonstrated [28]. Although the dissociation constant of the Aβ fibrils synthetized probe complex was only 0.395 mM-1, the significantly higher probe uptake was observed in AD rat brains compared to the healthy control brains. Additionally, an additional 19F NMR peak was observed from the AD brain. The authors hypothesized that this peak originated from the probe bounded to the Aβ plaques [28]. 

All molecular probes used for 19F MRI Aβ imaging and spectroscopy are gathered in Table 1.

### 2.2. Tau Pathology Imaging

**Figure 3:**
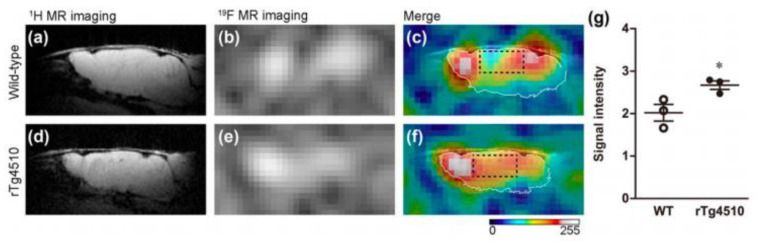
In vivo tau detection in wild-type (**a**–**c**) and rTg4510 (**d**–**f**) mice using 19F MRI after the injection of Shiga-X35 [32]. The proton MRI images (**a**,**d**) were used for brain localization. (**b**,**e**) 19F MRI images acquired after Shiga-X35 injection. Fluorine images were superimposed on top of high-resolution proton images (**c**,**f**). The Shiga-X35 uptake was significantly (* *p* = 0.044) higher in rTg4510 mice compared to the healthy controls (**g**). Images and graph are reprinted with permission from the publisher [32].

The aggregation of tau protein into neurofibrillary tangles (NFTs) represents a pathological hallmark of AD. Bundles of paired helical filaments (PHFs) comprise the NFTs and contain abnormally hyperphosphorylated tau in a β-sheet conformation [29,30,31]. It is known that tau NFT deposition correlates with cognitive decline in AD [4]. Thus, tau imaging within the brain can contribute to a minimally invasive diagnosis of AD, as well as a way to monitor disease activity and target engagement. The first study to report the detection of tau pathology using 19F-MRI employed the use of a styrl-benzoxazole derivative [32]. It has been previously reported that styrl-benzoxazole derivatives do not show binding to NFTs in AD brain sections [26]; however, compounds with a 15–18 Å of pi-electron-conjugated backbone show a high affinity to pathological tau [33]. Thus, to enhance the affinity of styrl-benzoxazole derivatives to tau pathologies, a double bond can be added to the pi-electron-conjugated backbone [32]. Recently, a buta-1,3-diene derivative, 1-(6-alkoxybenzoxazol-2-yl)-4-(4-dime-thylaminophenyl)-buta-1,3-dions (Shiga-X35), has been synthesized as a 19F MRI probe to detect NFTs. MR imaging of a live, transgenic mouse head after the injection of Shiga-X35 showed an accumulation of 19F-MR signals where NFTs accumulate in the brain, suggesting that the compound can be useful to detect tau pathology with a 7.0-T MR scanner (Figure 3). However, unwanted 19F-MR signals were detected in the wild-type mouse brain, demonstrating that the specificity and selectivity of the probe to NFTs should be improved. The sensitivity should also be improved, since a high dose of the probe was required (200 mg/kg), and the quality was poor compared to tau imaging using PET [32]. 

Studies have also investigated the ability of benzimidazole derivatives to selectively bind to NFTs [34]. Specifically, the use of lansoprazole (LNS), which has applications in the treatment of gastrointestinal disorders, can pass the blood-brain barrier and selectively bind to aggregated tau. Using surface plasmon resonance (SPR), LNS was shown to have a high affinity, indicated by a relatively low Ki, to PHFs isolated from AD brains (Ki = 833.3 ± 171 nM) [34]. Since LNS has a high density of fluorine within its structure, it can be useful in the application of tau imaging using 19F-MRI. Although these results did not show whether LNS can visualize tau pathology in the living brain, it suggests the use of fluorinated benzimidazole derivatives as potential probes for in vivo tau imaging. LNS was further used to detect tau aggregates in an ex vivo study using a rat model of AD and 19F-MRS. It was shown that the 19F signal from LNS interacts with NFTs, demonstrating the potential in using the compound to distinguish between AD and healthy brains with a clinical 3T scanner. The use of LNS as a fluorinated MRI molecular probe is advantageous, since the signal obtained mostly comes from pathology sites. Additionally, it is a commercially available product and already approved for medical use [35].

All molecular probes used for 19F MRI NFTs imaging and spectroscopy are gathered in Table 1.

## 3. 18F-Labeled PET Molecular Probes for AD Detection

### 3.1. Amyloid Beta Imaging

The use of PET is an important tool for Aβ imaging due to its high sensitivity and quantitative ability. Although MRI offers a higher spatial resolution, PET was the first imaging modality used for the molecular imaging of Aβ and NFTs. Furthermore, The National Institute on Aging-Alzheimer’s Association (NIA-AA) clinical diagnostic guidelines include amyloid PET biomarker data as a part of the diagnostic process [36,37]. The early reported use of radiolabeled amyloid molecular probes for potential in vivo PET imaging includes derivatives of Congo Red, chrysamine-G, and thioflavin T [38,39,40,41,42,43]. 

The first report to describe the in vivo detection of the pathological characteristics of AD (NFTs and Aβ) in the brain of living AD patients used 18F-labeled 2-(1-(6-[(2-(18F)fluoroethyl)(methyl)amino]-2-naphthyl)ethylidene)malononitrile ((18F)FDDNP). The probe crossed the blood-brain barrier in proportion to the blood flow and exhibited a greater accumulation and slower clearance from NFT and Aβ-dense brain regions [44]. In vitro studies indicated two kinetically distinguishable binding sites of (18F)FDDNP with Kd values of 0.12 nM-1 and 1.86 nM-1 to synthetic fibrils of Aβ(1–40) [45]. However, a study by Thompson et al. demonstrated that, when FDDNP is used at tracer concentrations associated with in vivo imaging studies, it has a low sensitivity for senile plaques and NFTs [46]. Following this, another human study was conducted using an 11C-labeled benzothiazole derivative, *N*-methyl-(11C)2-(4’-methylaminophenyl)-6-hydroxybenzothiazole (Pittsburgh Compound-B or PIB) and included 16 patients with AD and nine healthy volunteers. Compared to controls, this compound showed a marked retention of PIB in areas of the cortex known to show large amounts of amyloid depositions [47]. Further studies demonstrated the use of PIB-PET to detect Aβ in both AD patients and nondemented patients, suggesting its use at the preclinical stages of AD [48]. The major limitation of PIB includes its short half-life of 20 min, restricting its use to facilitates with an on-site cyclotron [47]. This triggered the study of further 18F-labeled radioligands for the imaging of Aβ in AD brains due to the longer half-life of 110 min. 

**Figure 4:**
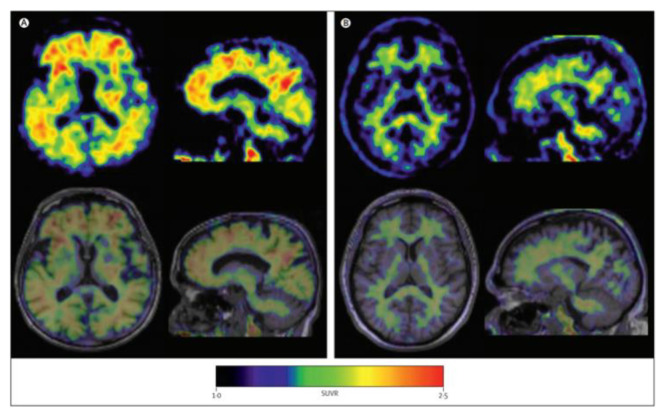
Axial and sagittal (18F)florbetaben brain PET images of an AD patient (**A**) and a healthy control (**B**) for the detection of Aβ [56]. The bottom row illustrates a fused PET/T1-weighted MRI image. Pet images were acquired 90–110 min post-injection. Images are reprinted with permission of the publisher [56].

Since then, newer 18F-labeled radiopharmaceuticals such as (18F)florbetapir (Amyvid; Eli Lilly, Indianapolis, IN, USA), (18F)florbetaben (Neuraceq; Piramal, Mumbai, India), and (18F)flutemetamol (Vizamyl; GE Healthcare, Arlington Heights, IL, USA) have been synthesized [49,50,51,52]. These compounds, all approved by the FDA, have a high affinity to Aβ plaques and can be used in the management of AD, as they allow for Aβ plaque quantification in brain cortices. These compounds have a half-life of 110 min, making them advantageous over PIB, since they can be transferred from the site of production to PET scanner facilities. [18F] Florbetapir, a stilbene derivative, was FDA-approved in 2012 and was the first nuclear imaging radiotracer to be used for subjects suspected with AD. It is readily taken up through the blood-brain barrier, and its biodistribution is stable up to 60 min after intravenous injection, allowing a wide window for the recommended imaging time of 10 min. Florbetapir PET images correlated with regions in the brain known to have Aβ [53]. (18F)florbetaben, another stilbene derivative, was FDA-approved in 2014 [54]. Preclinical data show a nanomolar binding affinity to both synthetic Aβ fibrils and AD homogenates without binding to tau or alpha-synuclein deposits, thus showing a high specificity to amyloid plaques [55]. In a phase II diagnostic study, florbetaben PET imaging differentiated healthy controls from AD patients with a sensitivity of 80% and a specificity of 91% (Figure 4) [56]. 

**Figure 5:**
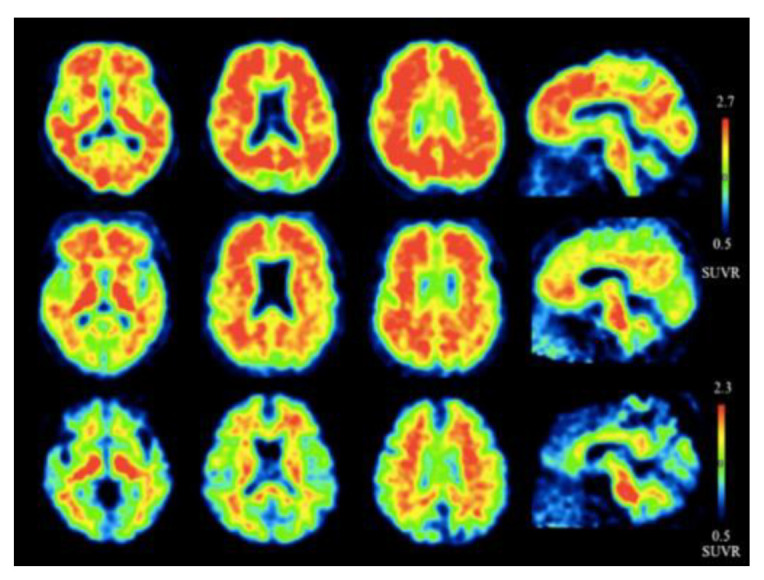
Representative axial and sagittal (18F)flutemetamol images of AD (upper row), mild cognitive impairment (middle row), and healthy controls (lower row) for the detection of Aβ [57]. Extensive uptake of flutemetamol can be observed in the cortical regions of AD and mild cognitive impairment patients. Images are reprinted with permission of the publisher [57].

Lastly, (18F)flutemetamol was FDA approved in 2013 and is an 18F derivative of PIB, thus demonstrating similar kinetic properties [54]. PET scans were able to distinguish healthy controls to AD patients with a sensitivity of 97.2% and specificity of 85.3% (Figure 5) [57]. 

Additional compounds used for Aβ imaging include 11C-labeled 5-(6-([tert-butyl(dimethyl)silyl]oxy)-1,3-benzothiazol-2-yl)pyridin-2-amine (11C-AZD2184), which has a high affinity for amyloid fibrils in vitro (Kd = 8.4 ± 1.0 nM). In human studies, a high binding of 11C-AZD2184 was observed in the cerebral cortical regions of AD patients in comparison with the control subjects [58]. All molecular probes used for 18F PET Aβ imaging are gathered in Table 2. 

### 3.2. Tau Pathology Imaging

**Figure 6:**
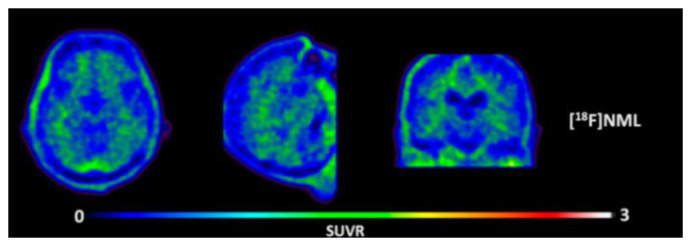
Representative transversal, sagittal, and coronal (18F)N-methyl lansoprazole (NML) PET images in a progressive supranuclear palsy patient for the detection of tau [70]. Images are reprinted with permission of the publisher [70].

Early radiotracers to detect tau pathology included quinolone and benzimidazole derivatives [59,60]. Of the quinolone derivatives, 2-(4-aminophenyl)-6-(2-(18F)fluoroethoxy) quinoline (18F-THK523) demonstrated a higher retention of the compound in the temporal, parietal, and orbitofrontal lobes and in the hippocampus of patients with AD, compared to healthy controls. However, the high retention of 18F-THK523 in white matter precludes a visual inspection of the images, hindering its use in clinical settings [61]. This led to improved quinoline derivatives, such as 6-((3-[18F]fluoro-2-hydroxy)propoxy)-2-(4-dimethylaminophenyl)quinolone (18F-THK5105), 6-((3-[18F] fluoro-2-hydroxy)propoxy)-2-(4-methylaminophenyl) quinolone (18F-THK5117), and 6-((3-18F-2-hydroxy)propoxy)-2-(4-methylaminopyridyl)quinoline (18F-THK5351), and showed a higher binding affinity than 18F-THK523 [60,62]. Of the three, 18F-THK5351 shows faster kinetics, a lower white matter retention, and a higher signal-to-noise ratio than 18F-THK5105 and 18F-THK5117 [60,62]. Benzimidazole and benzimidazole-pyrimidine derivatives include (E)-4-(2-(6-[2-(2-(2-[18F] fluoroethoxy) ethoxy) ethoxy] pyridin3-yl) vinyl)-*N*-methyl benzenamine (18F-T807, also known as 18F-flortaucipir) and lansoprazole for potential tau radiotracers [63,64,65]. Human 18F-T807 PET studies show high-target cortical-to-cerebellum ratios and accumulations in brain regions associated with the distribution of PHF-tau in AD brains [66]. However, in vivo studies demonstrate 18F-T807 to show nonspecific binding within the basal ganglia of AD and progressive supranuclear palsy patients [67]. This off-target binding may be attributed to 18F-T807 binding to monoamine oxidase (MAO) in the basal ganglia, as T807 is a weak, nonselective inhibitor of MAO [68]. A series of lansoprazole derivatives have been radiolabeled with 11C and 18F [64,69]. 11C-N-methyl lansoprazole (11C-NML) demonstrated a lack of brain uptake in mice but was overcome by pretreating with cyclosporin A to block the permeability-glycoprotein 1 (PGP) transporter. Contrastingly, both 11C-NML and 18F-lansoprazole demonstrated unhampered brain uptakes in nonhuman primates [64,69]. From docking studies, Rojo et al. demonstrated that the benzimidazole-NH formed a key hydrogen bond with the C-terminal hexapeptide (386TDHGAE391) of the tau core [34], thus determining that the effect of replacing the NH with N-11CH_3_ in 11C-NML is important. 11C-NML and nonlabeled lansoprazole had a Kd of 700 pM and 2.5 nM to heparin-induced tau filaments, respectively, suggesting that replacing the NH with N-11CH3 improves the affinity of tau by a factor of three [34,69]. More recently, the first-in-human study using 18F-NML as a tau PET imaging agent was evaluated (Figure 6). Despite the high affinity for tau in vitro, the brain retention in patients with AD progressive supranuclear palsy was low and insufficient to detect tau pathology in vivo [70]. 

Derivatives constructed around a phenyl/pyridinyl-butadienyl-benzothiazole/benzothiazolium (PBB) scaffold have been designed to detect tau pathologies within the brain. One of the first derivatives to be used, 11C-PBB3, was found to preferentially bind to NFTs in AD brains during an in vitro binding analysis than flortaucipir [71]. However, a low dynamic range, metabolic instability, and off-target binding in the basal ganglia were observed with the use of 11C-PBB3 [72]. Thus, 18F-labeled derivatives ((18F)AM-PBB3 and (18F)PM-PBB3) have been developed to overcome these drawbacks and demonstrated a greater signal-to-noise background ratio and less target binding within the basal ganglia [73,74].

After the development of the initial tau radiotracers discussed above, several pharmaceutical companies started developing and optimizing binding properties, leading to the second-generation of tau radiotracers. Some of these new tracers were based on the structure of existing tracers such as 18F-GTP1 and 18F-PI-2620 [75,76]. 18F-GTP1, a deuterated analog of 18F-T808, was synthesized to reduce the metabolic instability of 18F-T808 by defluorination. In a clinical PET study, 18F-GTP1 demonstrated a low nanomolar affinity to tau, selectivity over Aβ, no apparent off-target binding, and successfully differentiated AD subjects from healthy control subjects [75]. 18F-PI-2620, related to 18F-T807, was designed to reduce off-target binding towards MAO-A and -B. Using ligand-binding assays, 18F-PI-2620 demonstrated a high affinity to pathological tau aggregates and a selectivity for pathological tau aggregates over Aβ [76]. The first-in-human study using 18F-PI-2620 showed an accumulation of the radiotracer in regions of the brain known to have tau depositions, as well as increased uptakes of 18F-PI-2620 in patients with AD compared to healthy controls [77].

**Figure 7:**
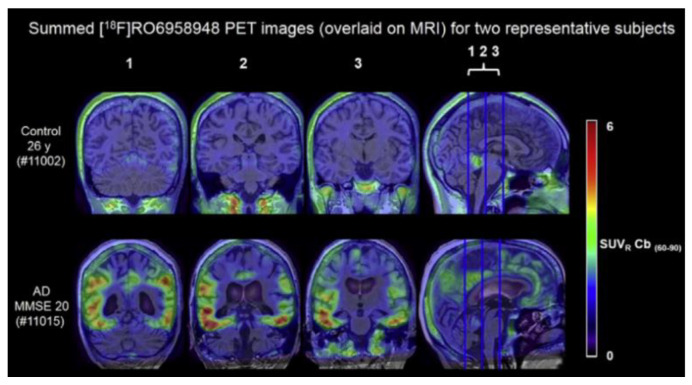
(18F)RO-948 PET images overlaid on MRI on a healthy control (upper row) and an AD patient (bottom row). The increased uptake of (18F)RO-948 in the AD patient is associated with the binding to the hyperphosphorylated tau protein. [80]. Images are reprinted with permission of the publisher [80].

Further radiotracers include 18F-RO6958948 (RO-948), 11C-RO6931643 (RO-643), and 11C-RO6924963 (RO-963) and were all identified to show a high affinity to NFTs and have selectivity against Aβ in AD brains [78,79]. Of the three, 18F-RO-948 had a better signal-to-background ratio in AD patients than 11C-RO-643 and 11C-RO-963. In a human study, a higher uptake of 18F-RO-948 was observed in AD patients compared to healthy controls (Figure 7). [80]. 18F-MK-6240, a pyridine isoquinoline amine derivative, is a novel radiotracer that demonstrates a high selectivity for NFTs [81]. In vivo studies in humans show 18F-MK-6240 to have a high affinity to tau without any substantial off-target binding in the basal ganglia that would impede NFT detection [82]. 18F-JNJ64349311 (18F-JNJ311) has also been shown to have a high affinity for aggregated tau (Ki= 8 nM) and in vivo selectivity for tau over Aβ [83]. 

All molecular probes used for 18F PET tau imaging are shown in Table 2.

## 4. Fluorescent Molecular Probes for AD Detection

### 4.1. Amyloid Beta Imaging

**Figure 8:**
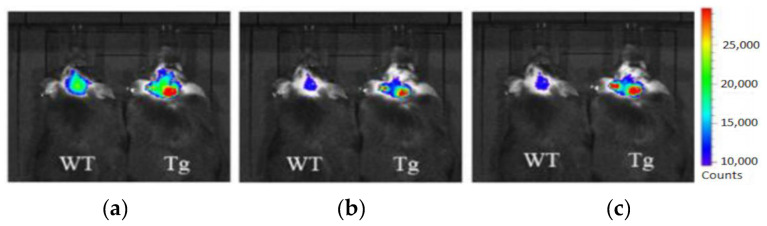
In vivo imaging of Aβ using QAD-1 in APPSWE/PSEN 1dE9 transgenic (Tg) and wild-type (WT) mice [90]. From left to right, images were taken 5 (**a**), 15 (**b**), and 30 (**c**) min after QAD-1 injection. The significant difference between the QAD-1 uptake in transgenic and wild-type mice was observed 5 min post-QAD-1 injection. The hot-spots on the images correspond to the Aβ plaques localized in the APPSWE/PSEN 1dE9 transgenic mouse. The low background signal of QAD-1 can be observed in (**c**). Images are reprinted with permission of the publisher [90].

Most research efforts for the molecular imaging of Aβ and NFTs have been focused on the use of MRI and PET. However, the sensitivity of MRI is low due to the small contrast between Aβ, NFTs, and surrounding tissue, while PET is limited by the short half-lives of the positron-emitting nuclei [84]. Alternative imaging modalities such as near-infrared fluorescence (NIRF) imaging depend on low absorption coefficients of light near the infrared region (600–900 nm) of the electromagnetic spectrum. These can be a powerful tool for the noninvasive imaging of specific pathological targets due to their relatively low cost, sensitivity, and the availability of dyes that fluoresce in this spectral domain [85]. Additionally, the fluorescence signals by the body within this range are minimal, resulting in low background signals [86]. One of the first NIRF probes used to image Aβ was NIAD-4 and was based off of the structures of Congo Red and thioflavin T. Although the probe was not fluorinated, it represents a major step in the development of additional NIRF ligands. In vitro studies demonstrated a binding constant of 10 nM, while in vivo studies demonstrated its ability to pass the blood-brain barrier and bind to Aβ. However, its emission wavelength falls just within the NIRF wavelength, and the technique used was still invasive [87]. Thus, further compounds were studied with improved optical properties and the ability to bind to Aβ. Several compounds have focused on the use of boron dipyrromethene (BODIPY) as a fluorophore for NIRF imaging due to its high quantum yield, biocompatibility, and high lipophilicity [15]. BODIPY-based Aβ aggregates (BAP)-1 contain a dimethylamino styryl group which plays an important role in Aβ aggregate binding. BAP-1 has a good affinity to synthetic Aβ fibrils in vitro, was able to penetrate the blood–brain barrier, and selectively labeled Aβ in a mouse brain. However, it failed in vivo imaging experiments due to nonspecific binding in the scalp [88]. Since the wavelengths of BODIPY derivatives can be increased by adding a furanyl group without affecting its ability to interact with Aβ, the dimethylaminophenyl group BAP-1 was replaced with a dimethylaminofuranyl group (BAP-2) [86,89]. Like BAP-1, BAP-2 was able to interact with Aβ in vitro but failed in vivo imaging due to nonspecific binding [86]. Thus, compounds such as QAD-1 have been synthesized in order to reduce nonspecific binding and reduce background signals. QAD-1 exhibited significant fluorescence once bound to soluble and insoluble Aβs with low background signals. In vivo studies demonstrated it could detect Aβ as early as six months in Aβ transgenic mice, suggesting that QAD-1 can be used for the early detection of AD (Figure 8) [90]. Other NIRF ligands include oxazine derivatives such as AOI987, which readily passed the blood-brain barrier, demonstrated a binding constant of 0.2 μM to aggregated Aβ, and has an absorption and emission maxima at 650 and 670 nm, respectively. In vivo studies revealed a higher fluorescence intensity in APP23-transgenic mice overexpressing human APP than age-matched healthy controls [85]. 

Due to its fluorescence properties, curcumin and its related derivatives have been investigated for Aβ plaque imaging using NIRF. It has been suggested that curcumin weakly passes the blood–brain barrier, as it took seven days of daily intravenous (I.V.) injections to observe the senile plaques [24]. Additionally, it is not practical to use for NIRF imaging due to its short emission wavelength [91]. However, a group of curcumin derivatives called CRANAD-Xs show better brain permeability than curcumin. Of the probes, CRANAD-2, -3, -58, and -102 show a high fluorescence increase when interacting with Aβ [92,93,94,95]. CRANAD-58 and -3 are used for the detection of both soluble and insoluble Aβ. This is favorable, since both forms coexist during AD progression [93,94]. CRANAD-102 was designed in order to differentiate between the insoluble and soluble forms of Aβ and demonstrated this 68-fold higher for soluble Aβ. CRANAD-102 can be used for the early detection of AD, since soluble Aβ is the dominant form in early stages of the disease [95]. Due to the poor tissue penetration of near-infrared (NIR) light, Yang et al. focused on the use of the eye as a target for noninvasive NIRF imaging, since it has minimal opacity for NIR light and is an extension of the central nervous system [96]. Studies have confirmed that Aβ species can deposit within the retina in transgenic mice [97], and humans express the amyloid precursor protein (APP) in the retina and have the cellular machinery to generate Aβ [98,99]. By using CRANAD-Xs, Aβ species were visualized using the near-infrared fluorescence ocular imaging (NIRFOI) of AD. However, the limitations of this technique include the inability to provide spatial resolution of the eye, the understanding of what brain areas are affected by Aβ, and the effects of the eye color of the NIRFOI signal [96]. 

Aβ is produced by two proteolytic events of APP, the first by β-site APP-cleaving enzyme 1 (BACE1). The use of fluorescence microscopy and a small molecule that can selectively bind to BACE1 can offer a way to study the physiology of this protease. SiR-BACE1, a conjugate of BACE1 inhibitor S-39, and silicone rhodamine (SiR)647 have been synthesized as a fluorogenic label for BACE1 and can be used for live cell imaging. The implications of SiR-BACE1 include the monitoring of AD therapeutics used to prevent BACE1–APP interactions [100].

All molecular probes used for fluorescence Aβ imaging are shown in Table 3.

### 4.2. Tau Pathology Imaging

Although thioflavin S (ThS) is the most common way to stain fibril structures in vitro, it is not applicable for in vivo studies, since it is unable to pass the blood–brain barrier due to its anionic nature [101]. Thus, attempts have been made to develop fluorescent compounds that have high selectivity for tau aggregates and pass the blood–brain barrier. Of these compounds, Congo red, difluoroboron, and BODIPY-based derivatives contain fluorine [102,103,104,105]. Of the Congo red derivatives, FSB was found to label tau inclusions in tissue sections from transgenic mice for human P301S tau. In vivo studies showed FSB to bind to the spinal cord of transgenic mice after a single intravenous injection [102].

**Figure 9:**
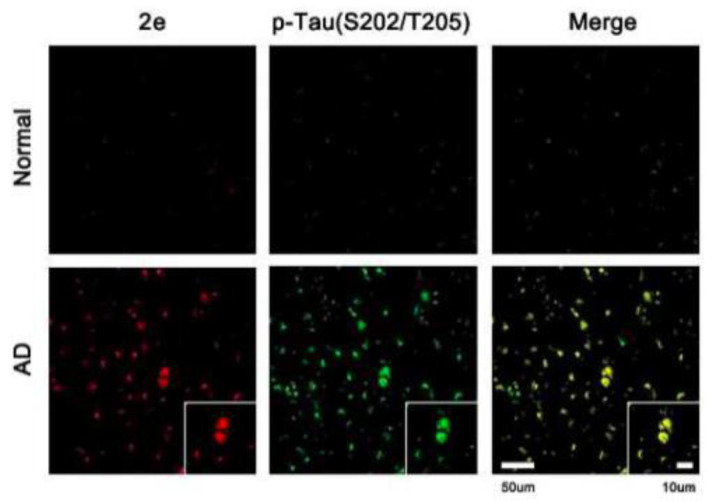
Confocal fluorescence images of healthy controls and AD brain sections after treatment with 6-((1E,3E)-4-(4-[dimethylamino]phenyl)-buta-1,3-dien-1-yl)-2,2-difluoro-4-319 isobutyl-2H-1,3,2-dioxaborinin-1-ium-2-uide to detect tau filaments (first column) [103]. The second column demonstrates staining with a *p*-tau antibody (Ser202/Thr205). The third column shows the superimposed images demonstrating the accuracy of t-protein detection using the difluoroboron β-diketonate probe. Images are reprinted with permission of the publisher [103].

Park et al. analyzed a series of variously substituted NIRF-specific tau probes with a difluoroboron β-diketonate and an N,N-32 dimethylaniline moiety linked by a length-extendable π-bridge [103]. Of these probes, 6-((1E,3E)-4-(4-[dimethylamino]phenyl)buta-1,3-dien-1-yl)-2,2-difluoro-4-319 isobutyl-2H-1,3,2-dioxaborinin-1-ium-2-uide (labeled 2e in the study) demonstrated 8.8-times higher tau specificity over Aβ, the highest of all probes investigated. Ex vivo studies showed AD brains to have a higher fluorescence than healthy controls (Figure 9). The probe demonstrates “turn-on” fluorescent properties due to the fluorophore with a rotatable donor-acceptor bond that selectively turns on in the microenvironment of tau aggregates [103].

**Figure 10:**
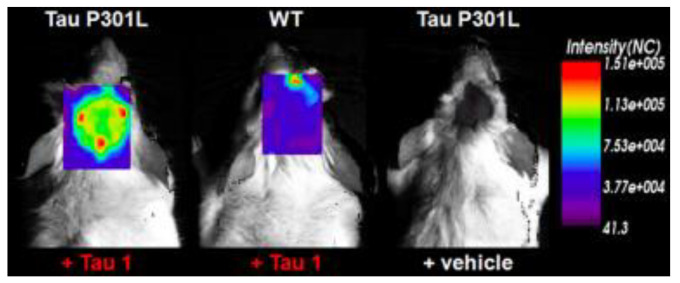
In vivo fluorescence imaging of a tau deposition in Tau P301L and wild-type mouse brain after the injection of either Tau 1 or the vehicle (negative control) [104]. The images were acquired using 670-nm excitation light 30 min after the injection of the molecular imaging probe. Images are reprinted with permission of the publisher [104].

BODIPY-based fluorescent probes have also been synthesized in order to image tau aggregates. Two of these probes, Tau 1 and Tau 2, demonstrated in vitro and ex vivo imaging of hyperphosphorylated tau filaments with low background noise. Additionally, both probes were able to visualize intracellular tau deposits, suggesting their ability to effectively penetrate cells. However, only Tau 1 was able to penetrate the blood–brain barrier and display a high selectivity for tau over Aβ in a transgenic mouse model (Figure 10) [104]. 

**Figure 11:**
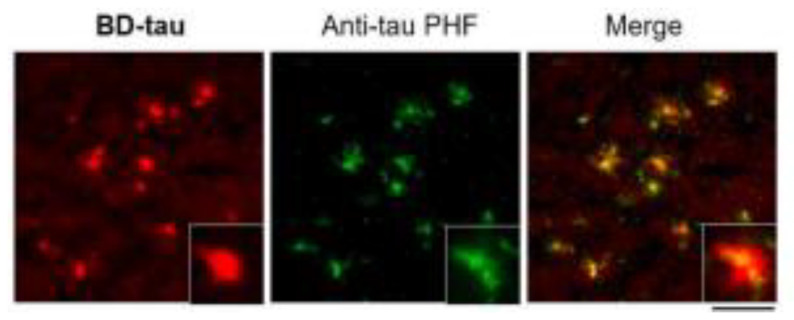
Ex vivo brain slices of tau transgenic mice labeled with the BD-tau and anti-tau paired helical filament (PHF) antibodies to demonstrate the accuracy of tau aggregates detection using BD-tau [105]. The third image is the overlay of the first two. Images are reprinted with permission of the publisher [105].

Additional BODIPY-based fluorescent probes include BD-tau and have been used to visualize tau aggregates in live cells. It was found that BD-tau was permeable to tau-induced cells and showed a 2.2-fold fluorescent increase upon interaction. Additionally, it was able to visualize tau aggregates in live cells but not under fixed-cell conditions. BD-tau was also able to detect filamentous tau aggregates in brain tissues, suggesting its use as a fluorescent probe in vivo (Figure 11) [105]. Since fluorescently labeling antibodies is expensive and time-consuming, fluorophores such as Tau-1, -2, and BD-tau can be advantageous in histological experiments [104].

All fluorinated molecular probes used for fluorescence tau imaging are demonstrated in Table 3.

## 5. Conclusions

Molecular imaging of the Aβ deposition and NFTs is crucial for the early detection and treatment monitoring of AD. Although the number of proposed fluorinated molecular probes is large, most of them have not reached the clinical translation stage. To facilitate future transitions into the clinic, molecular probe parameter optimizations are necessary. 

Molecular probes designed to bind to Aβ and hyperphosphorylated tau protein are small molecules comprised of at least two aromatic rings and a linker that may also serve to modify the probe’s affinity. For example, the vast majority of fluorinated molecular probes developed for AD detection contain either a methylamine or dimethylamine group that binds to π-conjugated structures, yielding a nanomolar affinity [61,92,106,107]. To make the MRI or PET probe detectable, it should be labeled using ^19^F or ^18^F, respectively. In order to develop fluorescent molecular imaging probes, molecules have to contain an electron-donating group linked to an electron-withdrawing group by a highly polarized conjugated π-electron chain. Alternating donor and acceptor groups can be used to control the relative energies of the highest occupied molecular orbital (HOMO) and the lowest unoccupied molecular orbital (LUMO). This leads to a smaller HOMO-LUMO gap and the desired long-wavelength absorption/emission bands [85,87]. Turning the compound’s fluorescence on upon binding to Aβ plaques can be achieved with the possession of a substantial degree of conformational freedom in the unbound state and restriction of the conformation after binding to the protein. This increases the fluorescence quantum yield on the protein-bound molecule by decreasing the vibration-rotational processes and, consequently, decreasing the radiationless decay rate [87].

The particular challenge that should be considered before synthesizing a novel fluorinated molecular imaging probe is the potentially high lipophilicity of the desired probe, which can limit the administrating concentrations. Thus, conjugating a hydrophilic group to improve the blood solubility should be considered. 

Two significant hydrophobic-binding sites located in Aβ_17–21_ and Aβ_30–40_ allow for hydrophobic and aromatic interactions with potential molecular probes. Thus, the aromatic core plays an important role in binding to Aβ [108]. Since Aβ and NFTs both exhibit a β-sheet structure, similar structural characteristics are used to design molecular probes specific for NFTs. Four different high-affinity binding sites have been observed on tau fibrils, with the dominant interaction for the probe-fibril association being hydrophobic [109]. 

Taking a close look at the affinity of all the studied fluorinated molecular probes, it can be noted that a vast majority have an association constant on the order of nM. A future development includes improving the probe’s association to the targeted protein structure. This approach involves precise biochemical calculations and modeling of the probe–target interactions. However, this approach is less beneficial for PET than MRI and fluorescence imaging, since the sensitivity of PET is extremely high, with a low spatial resolution. On the other hand, the MRI signal is directly proportional to the number of molecular probes at the disease site, and, therefore, a linear increase in the MRI signal with the probe’s affinity is expected. In addition, an increase of the probe affinity to their target decreases the background signal due to nonselective binding. Therefore, the signal-to-noise ratio of the MRI image is expected to increase even more. Currently, the major drawback of fluorinated MRI probes is the small number of ^19^F nuclei per molecule, which yields a low MRI signal and low image resolution. To eliminate this issue, increasing the amount of magnetically equivalent fluorine atoms per molecule is needed. This can be done either through attaching additional CF3 groups to the probe or through conjugating the probe to perfluorinated macromolecules, which are currently used to track the cell activity in vivo using MRI [110,111]. A higher affinity of fluorescence probes allows the reduction of the background signal due to nonspecific binding and, therefore, tends to increase the signal-to noise-ratio (SNR).

The other approach that potentially helps to improve the quality of the imaging of fluorinated AD molecular probes is the technical improvement of the signal detection systems. For example, the transition from whole-body PET to organ-specific PET can be beneficial by building a dedicated PET detector for brain imaging [112]. In addition, the development of novel image processing algorithms can significantly improve the resolution of PET imaging scans [113]. It can be expected that an improvement of the hardware and image processing software will be a key feature needed for the imaging of ^18^F-labeled PET molecular probes since PET imaging agents are highly developed, and the low resolution of PET images is limited by the positron traveling range. At the current stage of development, molecular imaging probes with low nonspecific binding should be preferred over other imaging probes. MRI imaging of fluorinated molecular probes can be improved by developing a dedicated phased-array brain coil that can substantially increase the SNR of an MRI image [114]. In addition, this approach can resolve a specific issue with all fluorinated MRI molecular probes—enormously long scanning time. Further advances can be achieved by using non-cartesian scanning k-space trajectories and the compressed sensing imaging approach [115]. These techniques will significantly decrease the scan time, improve the image SNR, and, therefore, improve the image resolution. 

Substantial improvement in the field of AD fluorescence imaging can be achieved by developing a probe with a large Stokes shift and high quantum yield. A large Stokes shift means the emission of the photons with a longer wavelength yielding to a better tissue penetration and reducing the fluorescence background.

**Figure 12:**
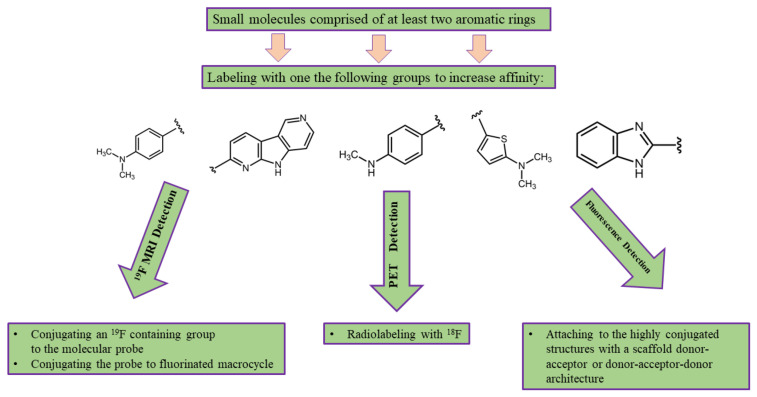
The suggested block scheme for the development of fluorinated molecular imaging probes for AD detection using one of the following medical imaging modalities: MRI, PET, and fluorescence imaging. Probes can be adjusted for single-photon emission computed tomography (SPECT) by radiolabeling with ^99m^Tc or ^131^I instead of ^18^F.

Depending on the imaging modality used, the desired properties of the molecular probe can be determined. For example, PET imaging probes should have a high affinity to the targeted protein but have a low nonspecific binding to decrease the background signal. MRI probes should contain a high number of ^19^F atoms per molecule, in addition to a high affinity and low nonselective binding. To make a proper decision about choosing a molecular probe for fluorescence imaging, molecules with the highest quantum yield and Stokes shift should be suggested. In addition, the changing of the fluorescence properties of the probe once bonded to the target can be a useful ability. Considering the desired properties of the molecular imaging probes, we can suggest the following block scheme (Figure 12) of developing an imaging probe for one of the three medical imaging modalities discussed above:

Overall, the early detection of AD with fluorinated molecular probes is an extensively developing field that has the potential to be translated into clinical practice. The successful clinical translation of fluorinated molecular probes can help increase the understanding of AD development, Aβ deposition, and NFT formation. These molecular probes can potentially help monitor AD treatment and, therefore, might be useful tools for AD drug development. In addition, these probes can facilitate early-stage AD detection, which makes them a valuable AD diagnostics instrument.

## Figures and Tables

**Table 1:** Molecular probes for the detection of amyloid-β (Aβ) or neurofibrillary tangles (NFTs) using 19F MRI.

Amyloid.	Tau
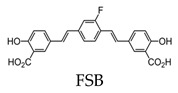	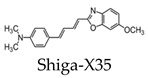
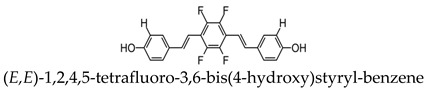	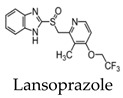
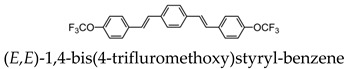	
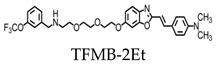	
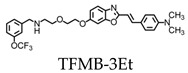	
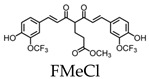	
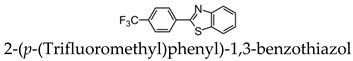	

**Table 2:** Molecular probes for the detection of Aβ or NFTs using PET.

Amyloid	Tau
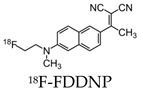	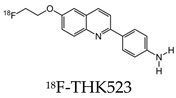
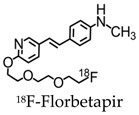	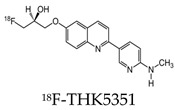
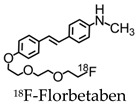	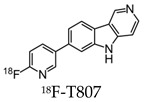
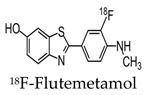	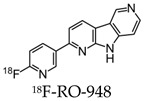
	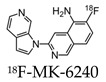
	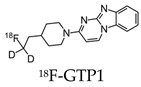
	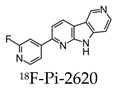
	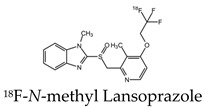

**Table 3:** Molecular probes for the detection of Aβ or NFTs using fluorescence. BAP: BODIPY-based Aβ aggregates.

Amyloid	Tau
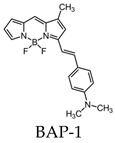	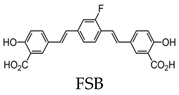
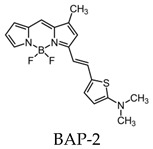	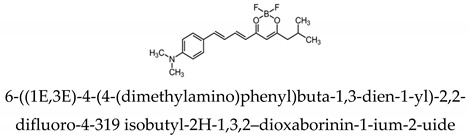
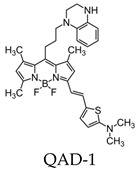	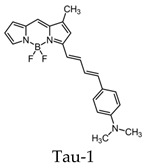
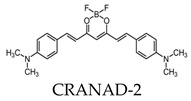	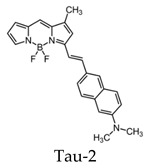
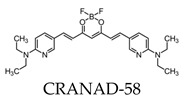	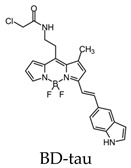
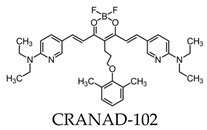	
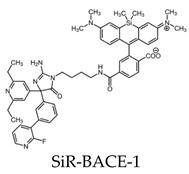	

## References

[1] Report A.A. (2020). 2020 Alzheimer’s disease facts and figures. Alzheimers Dement..

[2] Serrano-Pozo A., Frosch M.P., Masliah E., Hyman B.T. (2011). Neuropathological alterations in Alzheimer disease. Cold Spring Harb. Perspect. Med..

[3] Reitz C. (2012). Alzheimer’s disease and the amyloid cascade hypothesis: A critical review. Int. J. Alzheimers Dis..

[4] Giannakopoulos P., Herrmann F.R., Bussière T., Bouras C., Kövari E., Perl D.P., Morrison J.H., Gold G., Hof P.R. (2003). Tangle and neuron numbers, but not amyloid load, predict cognitive status in Alzheimer’s disease. Neurology.

[5] Deture M.A., Dickson D.W. (2019). The neuropathological diagnosis of Alzheimer’s disease. Mol. Neurodegener..

[6] Hane F.T., Robinson M., Lee B.Y., Bai O., Leonenko Z., Albert M.S. (2017). Recent Progress in Alzheimer’s Disease Research, Part 3: Diagnosis and Treatment. J. Alzheimers Dis..

[7] Gomes P.M.O., Silva A.M.S., Silva V.L.M. (2020). Pyrazoles as key scaffolds for the development of fluorine-18-labeled radiotracers for Positron Emission Tomography (PET). Molecules.

[8] Cherry S.R., Jones T., Karp J.S., Qi J., Moses W.W., Badawi R.D. (2018). Total-Body PET: Maximizing Sensitivity to Create New Opportunities for Clinical Research and Patient Care. J. Nucl. Med..

[9] Jagust W.J., Bandy D., Chen K., Foster N.L., Landau S.M., Mathis C.A., Price J.C., Reiman E.M., Skovronsky D., Koeppe R.A. (2010). The Alzheimer’s Disease Neuroimaging Initiative positron emission tomography core. Alzheimers Dement..

[10] Bagci U., Udupa J.K., Mendhiratta N., Foster B., Xu Z., Yao J., Chen X., Mollura D.J. (2013). Joint segmentation of anatomical and functional images: Applications in quantification of lesions from PET, PET-CT, MRI-PET, and MRI-PET-CT images. Med. Image Anal..

[11] Reitz C., Mayeux R. (2014). Alzheimer disease: Epidemiology, diagnostic criteria, risk factors and biomarkers. Biochem. Pharm..

[12] Frisoni G.B., Fox N.C., Jack C.R., Scheltens P., Thompson P.M. (2010). The clinical use of structural MRI in Alzheimer disease. Nat. Rev. Neurol..

[13] Ruiz-Cabello J., Barnett B.P., Bottomley P.A., Bulte J.W.M. (2011). Fluorine (19F) MRS and MRI in biomedicine. Nmr Biomed..

[14] Higuchi M., Iwata N., Matsuba Y., Sato K., Sasamoto K., Saido T.C. (2005). 19F and 1H MRI detection of amyloid β plaques in vivo. Nat. Neurosci..

[15] Tong H., Lou K., Wang W. (2015). Near-infrared fluorescent probes for imaging of amyloid plaques in Alzheimer’s disease. Acta Pharm. Sin. B.

[16] Hardy J.A., Higgins G.A. (1992). Alzheimer’s Disease: The Amyloid Alzheimer’s disease. Science.

[17] Hardy J., Selkoe D.J. (2002). The Amyloid Hypothesis of Alzheimer’s Disease: Progress and Problems on the Road to Therapeutics. Science.

[18] Flaherty D.P., Walsh S.M., Kiyota T., Dong Y., Ikezu T., Vennerstrom J.L. (2007). Polyfluorinated bis-styrylbenzene β-amyloid plaque binding ligands. J. Med. Chem..

[19] Amatsubo T., Morikawa S., Inubushi T., Urushitani M., Taguchi H., Shirai N., Hirao K., Kato M., Morino K., Kimura H. (2009). Trifluoromethoxy-benzylated ligands improve amyloid detection in the brain using 19F magnetic resonance imaging. Neurosci. Res..

[20] Yanagisawa D., Amatsubo T., Morikawa S., Taguchi H., Urushitani M., Shirai N., Hirao K., Shiino A., Inubushi T., Tooyama I. (2011). In vivo detection of amyloid β deposition using 19F magnetic resonance imaging with a 19F-containing curcumin derivative in a mouse model of Alzheimer’s disease. Neuroscience.

[21] McClure R., Yanagisawa D., Stec D., Abdollahian D., Koktysh D., Xhillari D., Jaeger R., Stanwood G., Chekmenev E., Tooyama I. (2015). Inhalable curcumin: Offering the potential for translation to imaging and treatment of Alzheimer’s disease. J. Alzheimers Dis..

[22] Yang F., Lim G.P., Begum A.N., Ubeda O.J., Simmons M.R., Ambegaokar S.S., Chen P., Kayed R., Glabe C.G., Frautschy S.A. (2005). Curcumin inhibits formation of amyloid β oligomers and fibrils, binds plaques, and reduces amyloid in vivo. J. Biol. Chem..

[23] Lim G.P., Chu T., Yang F., Beech W., Frautschy S.A., Cole G.M. (2001). The curry spice curcumin reduces oxidative damage and amyloid pathology in an Alzheimer transgenic mouse. J. Neurosci..

[24] Garcia-Alloza M., Borrelli L.A., Rozkalne A., Hyman B.T., Bacskai B.J. (2007). Curcumin labels amyloid pathology in vivo, disrupts existing plaques, and partially restores distorted neurites in an Alzheimer mouse model. J. Neurochem..

[25] Yanagisawa D., Shirai N., Amatsubo T., Taguchi H., Hirao K., Urushitani M., Morikawa S., Inubushi T., Kato M., Kato F. (2010). Relationship between the tautomeric structures of curcumin derivatives and their Aβ-binding activities in the context of therapies for Alzheimer’s disease. Biomaterials.

[26] Yanagisawa D., Taguchi H., Ibrahim N.F., Morikawa S., Shiino A., Inubushi T., Hirao K., Shirai N., Sogabe T., Tooyama I. (2014). Preferred features of a fluorine-19 MRI probe for amyloid detection in the brain. J. Alzheimers Dis..

[27] Yousaf M., Ahmad M., Bhatti I.A., Nasir A., Hasan M., Jian X., Kalantar-Zadeh K., Mahmood N. (2019). In Vivo and in Vitro Monitoring of Amyloid Aggregation via BSA@FGQDs Multimodal Probe. ACS Sens..

[28] Shepelytskyi Y., Campbell M.G., Hane F.T., Li T., Solomin V., Grynko V., Albert M.S. Fluorine-19 (19F) Labeled Benzothiazole Derivative as a Biosensor for detection of Alzheimer’s Disease using Magnetic Resonance Imaging. Proceedings of the International Society for Magnetic Resonance in Medicine.

[29] Ballatore C., Lee V.M.Y., Trojanowski J.Q. (2007). Tau-mediated neurodegeneration in Alzheimer’s disease and related disorders. Nat. Rev. Neurosci..

[30] Mandelkow E.M., Mandelkow E. (2012). Biochemistry and cell biology of Tau protein in neurofibrillary degeneration. Cold Spring Harb. Perspect. Med..

[31] Wang Y., Mandelkow E. (2016). Tau in physiology and pathology. Nat. Rev. Neurosci..

[32] Yanagisawa D., Ibrahim N.F., Taguchi H., Morikawa S., Kato T., Hirao K., Shirai N., Sogabe T., Tooyama I. (2017). Fluorine-19 magnetic resonance imaging probe for the detection of tau pathology in female rTg4510 mice. J. Neurosci. Res..

[33] Maruyama M., Shimada H., Suhara T., Shinotoh H., Ji B., Maeda J., Zhang M.R., Trojanowski J.Q., Lee V.M.Y., Ono M. (2013). Imaging of tau pathology in a tauopathy mouse model and in alzheimer patients compared to normal controls. Neuron.

[34] Rojo L.E., Alzate-Morales J., Saavedra I.N., Davies P., MacCioni R.B. (2010). Selective interaction of lansoprazole and Astemizole with tau polymers: Potential new clinical use in diagnosis of Alzheimers disease. J. Alzheimers Dis..

[35] Yeo S., Shepelytskyi Y., Grynko V., Hane F., Li T., Albert M. 19F MRS Detection of Tau Aggregates Using Lansoprazole in an Ex Vivo Rat Model of Alzheimer’s Disease. Proceedings of the International Society for Magnetic Resonance in Medicine.

[36] Albert M.S., DeKosky S.T., Dickson D., Dubois B., Feldman H.H., Fox N.C., Gamst A., Holtzman D.M., Jagust W.J., Petersen R.C. (2011). The diagnosis of mild cognitive impairment due to Alzheimer’s disease: Recommendations from the National Institute on Aging-Alzheimer’s Association workgroups on diagnostic guidelines for Alzheimer’s disease. Alzheimers Dement..

[37] McKhann G.M., Knopman D.S., Chertkow H., Hyman B.T., Jack C.R., Kawas C.H., Klunk W.E., Koroshetz W.J., Manly J.J., Mayeux R. (2011). The diagnosis of dementia due to Alzheimer’s disease: Recommendations from the National Institute on Aging-Alzheimer’s Association workgroups on diagnostic guidelines for Alzheimer’s disease. Alzheimers Dement..

[38] Klunk W.E., Debnath M.L., Pettegrew J.W. (1994). Development of small molecule probes for the Beta-amyloid protein of Alzheimer’s Disease. Neurobiol. Aging.

[39] Dezutter N.A., Dom R.J., De Groot T.J., Bormans G.M., Verbruggen A.M. (1999). 99mTC-MAMA-chrysamine G, a probe for beta-amyloid protein of Alzheimer’s disease. Eur. J. Nucl. Med..

[40] Agdeppa E.D., Kepe V., Liu J., Satyamurthy N., Barrio J.R. (2001). In vitro and in vivo binding characteristics of two biological probes for plaques and tangles in alzheimer’s disease. J. Label. Compd. Radiopharm..

[41] Klunk W.E., Wang Y., Huang G.F., Debnath M.L., Holt D.P., Mathis C.A. (2001). Uncharged thioflavin-T derivatives bind to amyloid-beta protein with high affinity and readily enter the brain. Life Sci..

[42] Mathis C.A., Bacskai B.J., Kajdasz S.T., McLellan M.E., Frosch M.P., Hyman B.T., Holt D.P., Wang Y., Huang G.F., Debnath M.L. (2002). A lipophilic thioflavin-T derivative for positron emission tomography (PET) imaging of amyloid in brain. Bioorganic Med. Chem. Lett..

[43] Mathis C.A., Wang Y., Holt D.P., Huang G.F., Debnath M.L., Klunk W.E. (2003). Synthesis and evaluation of 11C-labeled 6-substituted 2-arylbenzothiazoles as amyloid imaging agents. J. Med. Chem..

[44] Shoghi-Jadid K., Small G.W., Agdeppa E.D., Kepe V., Ercoli L.M., Siddarth P., Read S., Satyamurthy N., Petric A., Huang S.C. (2002). Localization of neurofibrillary tangles and beta-amyloid plaques in the brains of living patients with alzheimer disease. Am. J. Geriatr. Psychiatry.

[45] Agdeppa E.D., Kepe V., Liu J., Flores-Torres S., Satyamurthy N., Petric A., Cole G.M., Small G.W., Huang S.C., Barrio J.R. (2001). Binding characteristics of radiofluorinated 6-dialkylamino-2-naphthylethylidene derivatives as positron emission tomography imaging probes for beta-amyloid plaques in Alzheimer’s disease. J. Neurosci..

[46] Thompson P.W., Ye L., Morgenstern J.L., Sue L., Beach T.G., Judd D.J., Shipley N.J., Libri V., Lockhart A. (2009). Interaction of the amyloid imaging tracer FDDNP with hallmark Alzheimer’s disease pathologies. J. Neurochem..

[47] Klunk W.E., Engler H., Nordberg A., Wang Y., Blomqvist G., Holt D.P., Bergström M., Savitcheva I., Huang G.F., Estrada S. (2004). Imaging Brain Amyloid in Alzheimer’s Disease with Pittsburgh Compound-B. Ann. Neurol..

[48] Mintun M.A., Larossa G.N., Sheline Y.I., Dence C.S., Lee S.Y., MacH R.H., Klunk W.E., Mathis C.A., Dekosky S.T., Morris J.C. (2006). [11C]PIB in a nondemented population: Potential antecedent marker of Alzheimer disease. Neurology.

[49] Anand K., Sabbagh M. (2017). Amyloid Imaging: Poised for Integration into Medical Practice. Neurotherapeutics.

[50] Degenhardt E.K., Witte M.M., Case M.G., Yu P., Henley D.B., Hochstetler H.M., D’Souza D.N., Trzepacz P.T. (2016). Florbetapir F18 PET Amyloid Neuroimaging and Characteristics in Patients With Mild and Moderate Alzheimer Dementia. Psychosomatics.

[51] Daerr S., Brendel M., Zach C., Mille E., Schilling D., Zacherl M.J., Bürger K., Danek A., Pogarell O., Schildan A. (2017). Evaluation of early-phase [18F]-florbetaben PET acquisition in clinical routine cases. Neuroimage Clin..

[52] Lowe V.J., Lundt E., Knopman D., Senjem M.L., Gunter J.L., Schwarz C.G., Kemp B.J., Jack C.R., Petersen R.C. (2017). Comparison of [18F]Flutemetamol and [11C]Pittsburgh Compound-B in cognitively normal young, cognitively normal elderly, and Alzheimer’s disease dementia individuals. Neuroimage Clin..

[53] Clark C.M., Schneider J.A., Bedell B.J., Beach T.G., Bilker W.B., Mintun M.A., Pontecorvo M.J., Hefti F., Carpenter A.P., Flitter M.L. (2011). Use of florbetapir-PET for imaging β-amyloid pathology. JAMA J. Am. Med. Assoc..

[54] Filippi L., Chiaravalloti A., Bagni O., Schillaci O. (2018). 18F-labeled radiopharmaceuticals for the molecular neuroimaging of amyloid plaques in Alzheimer’s disease. Am. J. Nucl. Med. Mol. Imaging.

[55] Sabri O., Seibyl J., Rowe C., Barthel H. (2015). Beta-amyloid imaging with florbetaben. Clin. Transl. Imaging.

[56] Barthel H., Gertz H.J., Dresel S., Peters O., Bartenstein P., Buerger K., Hiemeyer F., Wittemer-Rump S.M., Seibyl J., Reininger C. (2011). Cerebral amyloid-β PET with florbetaben (18F) in patients with Alzheimer’s disease and healthy controls: A multicentre phase 2 diagnostic study. Lancet Neurol..

[57] Hatashita S., Yamasaki H., Suzuki Y., Tanaka K., Wakebe D., Hayakawa H. (2014). 18F Flutemetamol amyloid-beta PET imaging compared with [11C]PIB across the spectrum of Alzheimer’s disease. Eur. J. Nucl. Med. Mol. Imaging.

[58] Ito H., Shimada H., Shinotoh H., Takano H., Sasaki T., Nogami T., Suzuki M., Nagashima T., Takahata K., Seki C. (2014). Quantitative analysis of amyloid deposition in Alzheimer disease using PET and the radiotracer11C-AZD2184. J. Nucl. Med..

[59] Okamura N., Suemoto T., Furumoto S., Suzuki M., Shimadzu H., Akatsu H., Yamamoto T., Fujiwara H., Nemoto M., Maruyama M. (2005). Quinoline and benzimidazole derivatives: Candidate probes for in vivo imaging of tau pathology in Alzheimer’s disease. J. Neurosci..

[60] Okamura N., Furumoto S., Harada R., Tago T., Yoshikawa T., Fodero-Tavoletti M., Mulligan R.S., Villemagne V.L., Akatsu H., Yamamoto T. (2013). Novel 18F-labeled arylquinoline derivatives for noninvasive imaging of Tau pathology in Alzheimer disease. J. Nucl. Med..

[61] Villemagne V.L., Furumoto S., Fodero-Tavoletti M.T., Mulligan R.S., Hodges J., Harada R., Yates P., Piguet O., Pejoska S., Doré V. (2014). In vivo evaluation of a novel tau imaging tracer for Alzheimer’s disease. Eur. J. Nucl. Med. Mol. Imaging.

[62] Okamura N., Furumoto S., Fodero-Tavoletti M.T., Mulligan R.S., Harada R., Yates P., Pejoska S., Kudo Y., Masters C.L., Yanai K. (2014). Non-invasive assessment of Alzheimer’s disease neurofibrillary pathology using 18F-THK5105 PET. Brain.

[63] Xia C.F., Arteaga J., Chen G., Gangadharmath U., Gomez L.F., Kasi D., Lam C., Liang Q., Liu C., Mocharla V.P. (2013). [18F]T807, a novel tau positron emission tomography imaging agent for Alzheimer’s disease. Alzheimers Dement..

[64] Fawaz M.V., Brooks A.F., Rodnick M.E., Carpenter G.M., Shao X., Desmond T.J., Sherman P., Quesada C.A., Hockley B.G., Kilbourn M.R. (2014). High affinity radiopharmaceuticals based upon lansoprazole for PET imaging of aggregated tau in alzheimer"s disease and progressive supranuclear palsy: Synthesis, preclinical evaluation, and lead selection. ACS Chem. Neurosci..

[65] Harada R., Okamura N., Furumoto S., Furukawa K., Ishiki A., Tomita N., Tago T., Hiraoka K., Watanuki S., Shidahara M. (2016). 18F-THK5351: A novel PET radiotracer for imaging neurofibrillary pathology in Alzheimer disease. J. Nucl. Med..

[66] Chien D.T., Bahri S., Szardenings A.K., Walsh J.C., Mu F., Su M.Y., Shankle W.R., Elizarov A., Kolb H.C. (2013). Early Clinical PET Imaging Results with the Novel PHF-Tau Radioligand [F-18]-T807. J. Alzheimers Dis..

[67] Passamonti L., Rodríguez P.V., Hong Y.T., Allinson K.S.J., Williamson D., Borchert R.J., Sami S., Cope T.E., Bevan-Jones W.R., Jones P.S. (2017). 18F-AV-1451 positron emission tomography in Alzheimer’s disease and progressive supranuclear palsy. Brain.

[68] Drake L.R., Pham J.M., Desmond T.J., Mossine A.V., Lee S.J., Kilbourn M.R., Koeppe R.A., Brooks A.F., Scott P.J.H. (2019). Identification of AV-1451 as a Weak, Nonselective Inhibitor of Monoamine Oxidase. ACS Chem. Neurosci..

[69] Shao X., Carpenter G.M., Desmond T.J., Sherman P., Quesada C.A., Fawaz M., Brooks A.F., Kilbourn M.R., Albin R.L., Frey K.A. (2012). Evaluation of [11C] N-methyl lansoprazole as a radiopharmaceutical for PET imaging of tau neurofibrillary tangles. ACS Med. Chem. Lett..

[70] Kramer V., Brooks A.F., Haeger A., Kuljs R.O., Rafique W., Koeppe A., Raffel D.M., Frey K.A., Amaral H., Scott P.J.H. (2020). Evaluation of [F] N-methyl-lansoprazole as a tau PET imaging agent in first-in-human studies Evaluation of [18F] N-methyl-lansoprazole as a tau PET imaging agent in first-in- human studies. ACS Chem. Neurosci..

[71] Ono M., Sahara N., Kumata K., Ji B., Ni R., Koga S., Dickson D.W., Trojanowski J.Q., Lee V.M., Yoshida M. (2017). Distinct binding of PET ligands PBB3 and AV-1451 to tau fibril strains in neurodegenerative tauopathies. Brain.

[72] Villemagne V.L., Doré V., Burnham S.C., Masters C.L., Rowe C.C. (2018). Imaging tau and amyloid-β proteinopathies in Alzheimer disease and other conditions. Nat. Rev. Neurosci..

[73] Kitamura S., Ono M., Kimura Y., Ichise M., Takahata K., Morigucchi S., Kubota M., Ishii T., Takodo Y., Seki C. (2017). First -in-human PET study with 18F-AM-PBB3 and 18F-PM-PBB3. Alzheimers Dement..

[74] Wang Y.T., Edison P. (2019). Tau Imaging in Neurodegenerative Diseases Using Positron Emission Tomography. Curr. Neurol. Neurosci. Rep..

[75] Sanabria Bohórquez S., Marik J., Ogasawara A., Tinianow J.N., Gill H.S., Barret O., Tamagnan G., Alagille D., Ayalon G., Manser P. (2019). [18F]GTP1 (Genentech Tau Probe 1), a radioligand for detecting neurofibrillary tangle tau pathology in Alzheimer’s disease. Eur. J. Nucl. Med. Mol. Imaging.

[76] Kroth H., Oden F., Molette J., Schieferstein H., Capotosti F., Mueller A., Berndt M., Schmitt-Willich H., Darmency V., Gabellieri E. (2019). Discovery and preclinical characterization of [18F]PI-2620, a next-generation tau PET tracer for the assessment of tau pathology in Alzheimer’s disease and other tauopathies. Eur. J. Nucl. Med. Mol. Imaging.

[77] Mueller A., Bullich S., Barret O., Madonia J., Berndt M., Papin C., Perrotin A., Koglin N., Kroth H., Pfeifer A. (2019). Tau PET imaging with 18 F-PI-2620 in patients with Alzheimer’s disease and healthy controls: A first-in-human study. J. Nucl. Med..

[78] Gobbi L.C., Knust H., Körner M., Honer M., Czech C., Belli S., Muri D., Edelmann M.R., Hartung T., Erbsmehl I. (2017). Identification of Three Novel Radiotracers for Imaging Aggregated Tau in Alzheimer’s Disease with Positron Emission Tomography. J. Med. Chem..

[79] Honer M., Gobbi L., Knust H., Kuwabara H., Muri D., Koerner M., Valentine H., Dannals R.F., Wong D.F., Borroni E. (2018). Preclinical evaluation of 18F-RO6958948, 11C-RO6931643, and 11C-RO6924963 as novel PET radiotracers for imaging tau aggregates in Alzheimer disease. J. Nucl. Med..

[80] Wong D.F., Borroni E., Kuwabara H., Noble G., Rosenberg P.B., Lyketsos C., Resnik S., Thambisetty M., Brasic J., Gapasin L. (2018). First in-human PET study of 3 novel tau radiopharmaceuticals: [11C]RO6924963, [11C]RO6931643, and [18F]RO6958948. Alzheimers Dement..

[81] Walji A.M., Hostetler E.D., Selnick H., Zeng Z., Miller P., Bennacef I., Salinas C., Connolly B., Gantert L., Holahan M. (2016). Discovery of 6-(Fluoro-18F)-3-(1H-pyrrolo[2,3-c]pyridin-1-yl)isoquinolin-5-amine ([18F]-MK-6240): A Positron Emission Tomography (PET) Imaging Agent for Quantification of Neurofibrillary Tangles (NFTs). J. Med. Chem..

[82] Betthauser T., Cody K., Zammit M., Murali D., Converse A., Barnhart T., Stone C., Rowley H., Johnson S., Christian B. (2018). In vivo characterization and quantification of neurofibrillary tau PET radioligand [18F]MK-6240 in humans from Alzheimer’s disease dementia to young controls. J. Nucl. Med..

[83] Declercq L., Rombouts F., Koole M., Fierens K., Mariën J., Langlois X., Andrés J.I., Schmidt M., MacDonald G., Moechars D. (2017). Preclinical evaluation of 18F-JNJ64349311, a novel PET tracer for tau imaging. J. Nucl. Med..

[84] Staderini M., Martín M.A., Bolognesi M.L., Menéndez J.C. (2015). Imaging of β-amyloid plaques by near infrared fluorescent tracers: A new frontier for chemical neuroscience. Chem. Soc. Rev..

[85] Hintersteiner M., Enz A., Frey P., Jaton A.L., Kinzy W., Kneuer R., Neumann U., Rudin M., Staufenbiel M., Stoeckli M. (2005). In vivo detection of amyloid-β deposits by near-infrared imaging using an oxazine-derivative probe. Nat. Biotechnol..

[86] Watanabe H., Ono M., Matsumura K., Yoshimura M., Kimura H., Saji H. (2013). Molecular imaging of β-amyloid plaques with near-infrared boron dipyrromethane (BODIPY)-based fluorescent probes. Mol. Imaging.

[87] Nesterov E.E., Skoch J., Hyman B.T., Klunk W.E., Bacskai B.J., Swager T.M. (2005). In Vivo Optical Imaging of Amyloid Aggregates in Brain: Design of Fluorescent Markers. Angew. Chem..

[88] Ono M., Watanabe H., Kimura H., Saji H. (2012). BODIPY-based molecular probe for imaging of cerebral β-amyloid plaques. ACS Chem. Neurosci..

[89] Lee J.S., Kang N.Y., Yun K.K., Samanta A., Feng S., Hyeong K.K., Vendrell M., Jung H.P., Chang Y.T. (2009). Synthesis of a BODIPY library and its application to the development of live cell glucagon imaging probe. J. Am. Chem. Soc..

[90] Ren W., Zhang J., Peng C., Xiang H., Chen J., Peng C., Zhu W., Huang R., Zhang H., Hu Y. (2018). Fluorescent Imaging of β-Amyloid Using BODIPY Based Near-Infrared Off-On Fluorescent Probe. Bioconj. Chem..

[91] Ryu E.K., Choe Y.S., Lee K.H., Choi Y., Kim B.T. (2006). Curcumin and dehydrozingerone derivatives: Synthesis, radiolabeling, and evaluation for β-amyloid plaque imaging. J. Med. Chem..

[92] Ran C., Xu X., Raymond S.B., Ferrara B.J., Neal K., Bacskai B.J., Medarova Z., Moore A. (2009). Design, synthesis, and testing of difluoroboron-derivatized curcumins as near-infrared probes for in vivo detection of amyloid-β deposits. J. Am. Chem. Soc..

[93] Zhang X., Tian Y., Li Z., Tian X., Sun H., Liu H., Moore A., Ran C. (2013). Design and synthesis of curcumin analogues for in vivo fluorescence imaging and inhibiting copper-induced cross-linking of amyloid beta species in alzheimer’s disease. J. Am. Chem. Soc..

[94] Zhang X., Tian Y., Zhang C., Tian X., Ross A.W., Moir R.D., Sun H., Tanzi R.E., Moore A., Ran C. (2015). Near-infrared fluorescence molecular imaging of amyloid beta species and monitoring therapy in animal models of Alzheimer’s disease. Proc. Natl. Acad. Sci. USA.

[95] Li Y., Yang J., Liu H., Yang J., Du L., Feng H., Tian Y., Cao J., Ran C. (2017). Tuning the stereo-hindrance of a curcumin scaffold for the selective imaging of the soluble forms of amyloid beta species. Chem. Sci..

[96] Yang J., Yang J., Li Y., Xu Y., Ran C. (2019). Near-infrared Fluorescence Ocular Imaging (NIRFOI) of Alzheimer’s Disease. Mol. Imaging Biol..

[97] Ning A., Cui J., To E., Ashe K.H., Matsubara J. (2008). Amyloid-β deposits lead to retinal degeneration in a mouse model of Alzheimer disease. Investig. Ophthalmol. Vis. Sci..

[98] Johnson L.V., Leitner W.P., Rivest A.J., Staples M.K., Radeke M.J., Anderson D.H. (2002). The Alzheimer’s Aβ-peptide is deposited at sites of complement activation in pathologic deposits associated with aging and age-related macular degeneration. Proc. Natl. Acad. Sci. USA.

[99] Ratnayaka J.A., Serpell L.C., Lotery A.J. (2015). Dementia of the eye: The role of amyloid beta in retinal degeneration. Eye.

[100] Karch S., Broichhagen J., Schneider J., Böning D., Hartmann S., Schmid B., Tripal P., Palmisano R., Alzheimer C., Johnsson K. (2018). A New Fluorogenic Small-Molecule Labeling Tool for Surface Diffusion Analysis and Advanced Fluorescence Imaging of β-Site Amyloid Precursor Protein-Cleaving Enzyme 1 Based on Silicone Rhodamine: SiR-BACE1. J. Med. Chem..

[101] Gao X., Wang L., Huang H.L., Wang L.L., Yao J.L., Shi S., Yao T.M. (2015). Molecular “light switch” [Ru(phen)2dppzidzo]^2+^ monitoring the aggregation of tau. Analyst.

[102] Velasco A., Fraser G., Delobel P., Ghetti B., Lavenir I., Goedert M. (2008). Detection of filamentous tau inclusions by the fluorescent Congo red derivative FSB [(trans,trans)-1-fluoro-2,5-bis(3-hydroxycarbonyl-4-hydroxy)styrylbenzene]. Febs Lett..

[103] Park K.S., Kim M.K., Seo Y., Ha T., Yoo K., Hyeon S.J., Hwang Y.J., Lee J., Ryu H., Choo H. (2017). A Difluoroboron β-Diketonate Probe Shows “turn-on” Near-Infrared Fluorescence Specific for Tau Fibrils. ACS Chem. Neurosci..

[104] Verwilst P., Kim H.R., Seo J., Sohn N.W., Cha S.Y., Kim Y., Maeng S., Shin J.W., Kwak J.H., Kang C. (2017). Rational Design of in Vivo Tau Tangle-Selective Near-Infrared Fluorophores: Expanding the BODIPY Universe. J. Am. Chem. Soc..

[105] Lim S., Haque M.M., Su D., Kim D., Lee J.S., Chang Y.T., Kim Y.K. (2017). Development of a BODIPY-based fluorescent probe for imaging pathological tau aggregates in live cells. Chem. Commun..

[106] Biancalana M., Koide S. (2010). Molecular mechanism of Thioflavin-T binding to amyloid fibrils. Biochim. Biophys. Acta.

[107] Yang Y., Cui M. (2014). Radiolabeled bioactive benzoheterocycles for imaging β-amyloid plaques in Alzheimer’s disease. Eur. J. Med. Chem..

[108] Duan X.H., Liu B.L. (2008). Aβ-binding molecules: Possible application as imaging probes and as anti-aggregation agents. Sci. China Ser. B Chem..

[109] Murugan N.A., Nordberg A., Ågren H. (2018). Different Positron Emission Tomography Tau Tracers Bind to Multiple Binding Sites on the Tau Fibril: Insight from Computational Modeling. ACS Chem. Neurosci..

[110] Chirizzi C., De Battista D., Tirotta I., Metrangolo P., Comi G., Bombelli F.B., Chaabane L. (2019). Multispectral MRI with Dual Fluorinated Probes to Track Mononuclear Cell Activity in Mice. Radiology.

[111] Jirak D., Galisova A., Kolouchova K., Babuka D., Hruby M. (2019). Fluorine polymer probes for magnetic resonance imaging: Quo vadis?. Magn. Reson. Mater. Phys. Biol. Med..

[112] Gonzalez A.J., Sanchez F., Benlloch J.M. (2018). Organ-Dedicated Molecular Imaging Systems. IEEE Trans. Radiat. Plasma Med. Sci..

[113] Poladyan H., Bubon O., Teymurazyan A., Senchurov S., Reznik A. (2020). Gaussian position weighted center of gravity algorithm for multiplexed readout. Phys. Med. Biol..

[114] Hamilton J., Franson D., Seiberlich N. (2017). Recent advances in parallel imaging for MRI. Prog. Nucl. Magn. Reson. Spectrosc..

[115] Feng L., Benkert T., Block K.T., Sodickson D.K., Otazo R., Chandarana H. (2017). Compressed sensing for body MRI. J. Magn. Reson. Imaging.

